# An inter-laboratory study to investigate the impact of the bioinformatics component on microbiome analysis using mock communities

**DOI:** 10.1038/s41598-021-89881-2

**Published:** 2021-05-19

**Authors:** Denise M. O’Sullivan, Ronan M. Doyle, Sasithon Temisak, Nicholas Redshaw, Alexandra S. Whale, Grace Logan, Jiabin Huang, Nicole Fischer, Gregory C. A. Amos, Mark D. Preston, Julian R. Marchesi, Josef Wagner, Julian Parkhill, Yair Motro, Hubert Denise, Robert D. Finn, Kathryn A. Harris, Gemma L. Kay, Justin O’Grady, Emma Ransom-Jones, Huihai Wu, Emma Laing, David J. Studholme, Ernest Diez Benavente, Jody Phelan, Taane G. Clark, Jacob Moran-Gilad, Jim F. Huggett

**Affiliations:** 1 Molecular Biology, National Measurement Laboratory, LGC, Queens Road, Teddington, TW11 0LY Middlesex UK; 2 Department of Microbiology, Virology and Infection Control, Great Ormond Street Hospital for Children NHS Trust, Great Ormond Street, London, WC1N 3JH UK; 3 Department of Infection, Immunity and Inflammation, UCL Great Ormond Street Institute of Child Health and Reubens Centre of Paediatric Virology and Metagenomics, 30 Guildford Street, London, WC1N EH UK; 4 Institute of Medical Microbiology, Virology and Hygiene, University Medical Center Hamburg-Eppendorf, UKE, Martinstraße 52, 20246 Hamburg, Germany; 5 Department of Bacteriology, TDI, National Institute for Biological Standards and Control, South Mimms, EN6 3QG UK; 6 School of Biosciences, Cardiff University, Cardiff, CF10 3AX UK; 7 Division of Digestive Diseases, Imperial College London, London, UK; 8 Pathogens and Microbes, Wellcome Sanger Institute, Wellcome Genome Campus, Hinxton, Cambridge, CB10 1SA UK; 9 Department of Health System Management, School of Public Health, Faculty of Health Sciences, Ben-Gurion University of the Negev, Negev, Israel; 10 European Molecular Biology Laboratory, European Bioinformatics Institute (EMBL-EBI), Wellcome Trust Genome Campus, Hinxton, Cambridge, CB10 1SD UK; 11 Medical Microbiology Research Laboratory, Bob Champion Research and Educational Building, University of East Anglia, Norwich, NR4 7UQ UK; 12 Quadram Institute Bioscience, Norwich Research Park, Norwich, NR4 7UA UK; 13 Department of Biological and Geographical Sciences, School of Applied Sciences, University of Huddersfield, Huddersfield, HD1 3DH UK; 14 School of Biosciences, University of Surrey, Guildford, GU2 7XH UK; 15 Biosciences, University of Exeter, Stocker Road, Exeter, EX4 4QD UK; 16 Faculty of Infectious and Tropical Diseases, London School of Hygiene and Tropical Medicine, London, WC1E 7HT UK; 17 Faculty of Epidemiology and Population Health, London School of Hygiene and Tropical Medicine, London, WC1E 7HT UK; 18 School of Biosciences and Medicine, Faculty of Health and Medical Sciences, University of Surrey, Guildford, GU2 7XH UK; 19 Present Address: Department of Veterinary Medicine, University of Cambridge, Madingley Road, Cambridge, CB3 0ES UK; 20 Present Address: Department of Genetics, University of Cambridge, Cambridge, UK; 21 Present Address: Victorian Infectious Disease Reference Laboratory, Peter Doherty Institute for Infection and Immunity, Melbourne, 3000 Australia

**Keywords:** Sequencing, Metagenomics

## Abstract

Despite the advent of whole genome metagenomics, targeted approaches (such as 16S rRNA gene amplicon sequencing) continue to be valuable for determining the microbial composition of samples. Amplicon microbiome sequencing can be performed on clinical samples from a normally sterile site to determine the aetiology of an infection (usually single pathogen identification) or samples from more complex niches such as human mucosa or environmental samples where multiple microorganisms need to be identified. The methodologies are frequently applied to determine both presence of micro-organisms and their quantity or relative abundance. There are a number of technical steps required to perform microbial community profiling, many of which may have appreciable precision and bias that impacts final results. In order for these methods to be applied with the greatest accuracy, comparative studies across different laboratories are warranted. In this study we explored the impact of the bioinformatic approaches taken in different laboratories on microbiome assessment using 16S rRNA gene amplicon sequencing results. Data were generated from two mock microbial community samples which were amplified using primer sets spanning five different variable regions of 16S rRNA genes. The PCR-sequencing analysis included three technical repeats of the process to determine the repeatability of their methods. Thirteen laboratories participated in the study, and each analysed the same FASTQ files using their choice of pipeline. This study captured the methods used and the resulting sequence annotation and relative abundance output from bioinformatic analyses. Results were compared to digital PCR assessment of the absolute abundance of each target representing each organism in the mock microbial community samples and also to analyses of shotgun metagenome sequence data. This ring trial demonstrates that the choice of bioinformatic analysis pipeline alone can result in different estimations of the composition of the microbiome when using 16S rRNA gene amplicon sequencing data. The study observed differences in terms of both presence and abundance of organisms and provides a resource for ensuring reproducible pipeline development and application. The observed differences were especially prevalent when using custom databases and applying high stringency operational taxonomic unit (OTU) cut-off limits. In order to apply sequencing approaches with greater accuracy, the impact of different analytical steps needs to be clearly delineated and solutions devised to harmonise microbiome analysis results.

## Introduction

The analysis of the microbial composition of a sample can be performed using high-throughput sequencing methods by amplifying and sequencing selected regions of a gene (a metagenetic approach) or whole metagenomic DNA. Amplicon sequencing of regions such as the 16S ribosomal RNA gene (16S rRNA) continue to provide a simplified approach that is widely applied for bacterial identification and microbial community profiling^1–3^ even though there are newer approaches which involve sequencing of whole metagenomes. This is in part due to being an order of magnitude cheaper compared to a shotgun metagenomic approach while also being able to cope with the presence of a high background of contaminating (e.g. human) genomic DNA. In addition, targeted approaches require less computing power, and are well established, so have more complete, actively maintained and extensive databases and highly developed workflows. In this approach, highly conserved regions of the 16S rRNA gene are most often chosen as PCR primer binding sites to span variable region(s) that provide sequence clustering at the level of Operational Taxonomic Units (OTU). While this strategy is widely used, conserved regions of the 16S rRNA gene are not universally conserved across all microbial taxa^4^, and this sequence variability at primer-binding sites causes bias in microbial profiling experiments^5,6^. These biases can be further driven by the variable amplification efficiencies of different primer sets due to template-primer mismatches which will further distort the abundances of certain taxa when observing microbial community structure^7^. Conversely, shotgun metagenomic sequencing, which does not require sequence-dependent primer annealing, is thought to introduce less bias especially if it is prepared without PCR^8^.

There are many technical steps required in performing 16S rRNA gene amplicon sequencing experiments that can influence the results^9^. These include sampling (sampling site, method, sample transport and storage), extraction of nucleic acid material, choice of 16S rRNA primer, amplification, library preparation, sequencing and bioinformatic analysis pipeline. Previously, we used control materials (*i.e.*, defined mock communities of mixed organism nucleic acids) to investigate how different steps in the process impact on the observed results^10,11^. Other studies have investigated how DNA extraction methods^12–14^, sample storage^14^, and variable 16S rRNA gene copy number can impact observed microbial community structures^15^. Hiergeist et al. used an inter-laboratory study to evaluate 16S rRNA gene amplicon sequencing of stool samples^16^. They concluded that investigators need to perform proficiency testing as all steps of the workflow can significantly affect the output of the procedure. However, the study did not evaluate the impact of the bioinformatic approach on error in isolation. Other studies have used simulated data sets to evaluate the bioinformatics approach^17^.

The use of control materials can enable the measurement of technical error. We previously investigated the application of a control material^10^ to investigate factors including choice of 16S rRNA gene amplicon strategy and impact of sequencing depth on whole genome sequencing data^11^. In that study, the control materials were characterised by absolute quantification of each organism contained in the mixture using a method orthogonal to sequencing called digital PCR (dPCR). dPCR is a highly accurate method for absolute quantification of DNA targets in samples^18,19^, and can be applied as a primary reference measurement procedure^20^. Previous studies have demonstrated that the choice of bioinformatic approach for analysis of microbiome sequence data can strongly impact the inferred microbial taxa composition^21^ and observed taxon relative abundance^22^. There are multiple decision points in bioinformatics pipelines, including quality filtering method, chimera removal method, 16S rRNA database choice, alignment method and assignment of taxonomy methodology. All of these steps can be performed by pipeline tools such as QIIME (Quantitative Insights Into Microbial Ecology)^23^, MG-RAST (Metagenomics Rapid Annotations using Subsystems Technology)^24,25^, MGnify^26^ and mothur^27^ or these tools can be combined with customised pipelines for the analysis of data. In addition to the broad choice of bioinformatic approaches, each tool has alterable parameters which affect the outcome. All of these options increase analysis variability, so it is important that a comprehensive description of data processing steps is included in the methodology. More stringent requirements in data reporting via a guideline system such as the Minimum Information about any (X) Sequence (MIxS) requirements for publishing will help^28^.

Inter-laboratory studies are a way to determine the reproducibility of methods and this is especially important in the field of multi-step advanced sequence analysis. In order to apply these methods with confidence, pipelines need to be validated through verification of inter-laboratory agreement using mock communities, as demonstrated in our previous study^11^. Few studies to date have investigated the reproducibility of results. Prior multi-centre studies have focused on next generation sequence based oncology tests^29^, detection of complex variants^30^ and whole genome sequence (WGS) based bacterial genotyping^31^. Other studies have looked into the benchmarking of 16S rRNA gene amplicon sequencing data^32^, investigating the tools for clustering of data in microbiome studies^33^ and making resources for benchmarking of data publicly available^34^. In addition to these studies, there are initiatives from the Global Microbial Identifier (GMI) initiative which has performed proficiency testing schemes for bacterial isolates^35,36^, and the European Molecular Genetics Quality Network (EMQN) which runs external quality assessment (EQA) schemes for germline and somatic mutation testing.

In this study, we used an inter-laboratory comparison to investigate the impact of the bioinformatic processing step on the prediction of the composition of control materials (*i.e.*, genomic DNA from mock community samples, MCM2α and MCM2β). Raw sequence data, generated from PCR-next-generation amplicon sequencing of different 16S rRNA gene variable regions, which included technical repeats, were shared with multiple laboratories for bioinformatic pipeline comparison.

## Methods

### Preparation of Metagenomic Control Materials (MCM) MCM2α and MCM2β

The metagenomic control material (MCM) 2α and β contained 15 different bacterial and one viral species, representing common human pathogens (Table 1). The two materials varied only by one species of *Enterococcus* but there were also subtle differences in the quantity of each organism in the mixture. This design was implemented in order to interrogate the ability of the sequencing approaches to identify these subtle differences.

**Table 1:** The composition of the MCM2α and MCM2β.

MCMα	MCMβ	ATCC product code
*Staphylococcus aureus* (Methicillin sensitive MSSA)	MSSA	BAA-1718D-5
*S. aureus* (Methicillin resistant MRSA)	*–*	BAA-1556D-5
	*Staphylococcus epidermidis* (Methicillin Resistant MRSE)	35984D-5
*Streptococcus pneumoniae (PBP2B)*	*–*	700669D-5
	*S. pneumoniae*	33400D-5
*Streptococcus pyogenes*	*S. pyogenes*	700294D-5
*Streptococcus agalactiae*	*S. agalactiae*	BAA-611D-5
*Enterococcus faecalis*		700802D-5
*–*	*Enterococcus faecium*	BAA-472D-5
*–*	*E. faecium* (Vancomycin resistant—VRE)	51559D-5
*Pseudomonas aeruginosa*	*P. aeruginosa*	47085D-5
*Klebsiella pneumoniae*	*K. pneumoniae*	700721D-5
*Acinetobacter baumannii*	*A .baumannii*	17978D-5
*Escherichia coli*	*–*	700928D-5
*–*	*E. coli (O157:H7)*	IRMM-449
*Neisseria meningitidis*	*N. meningitidis*	700532D-5
*Moraxella catarrhalis*	*M. catarrhalis*	25240D-5
*Haemophilus influenzae*	*H. influenzae*	51907D
*Mycobacterium tuberculosis*	*M. tuberculosis*	25618D-2
Human Cytomegalovirus	hCMV	VR-538D
*Salmonella enterica*	*S*. *enterica*	700720D-5

The materials were prepared using genomic DNA (gDNA) sourced from ATCC (LGC Standards) and gDNA from *E. coli* O157, strain EDL 933 from IRMM (Institute for Reference Materials and Measurements). The concentration (ng/µL) of each gDNA preparation was determined by observing the mean value using triplicate measurements using a Qubit dsDNA BR Assay Kit (ThermoFisher) on the Qubit Fluorometer. The concentration of each gDNA preparation was also determined using specific assays for each of the organisms (Table 2) in the materials using dPCR using methods previously developed^11^. dPCR analyses were performed on a Bio-Rad QX200 droplet digital PCR system. This value was used when preparing the materials. The materials were diluted in TE pH 7.0 buffer and incubated at 4 °C for 4 h on a tube rotator. Aliquots of the materials were prepared in a final volume of 25 µL and stored at − 80 °C. Stability of the materials was determined as previously described^11^ using assays *ctrA* amplifying *N. meningitidis* and *hin* from MCM2α, *mor* and *lytA* from MCM2β. The material composition was determined using microfluidic dPCR as previously described^11^ using the assays in Table 2. The dMIQE (Minimum Information for publication of Quantitative Digital PCR Experiments) checklist is included in Additional File 1.

**Table 2:** List of gene targets and corresponding assays for quantifying materials.

Organism	Gene	Accession no.	Oligonucleotide Name	Sequence (5′–3′)	Amplicon size (bp)	References
*A. baumannii*	*ompA*	KJ363323	ompA_F	CATGGAACTTCGTGTGATTCTTTG	111	O’Sullivan et al. (2014)^14^
			ompA_R	GCAGTAGCGTTAGGGTATTCAGATAAT		
			ompA_MGB	[6FAM] AAATCAAACATCAAAGACC [MGBNFQ]		
*E. coli*	*uidA*	AE014075	uidA_F	GCCCGCTTCGAAACCAAT	120	O’Sullivan et al. (2014)^14^
			uidA_R	TCGCATTACCCTTACGCTGAA		
			uidA_HP	[6FAM] TCCATGTTCATCTGCCCAGTCGAGC [BHQ1]		
*E. faecalis*	*groES*	AF335185	groES_F	TTACTGTGTCACCAATTTTTACTTCCA	96	O’Sullivan et al. (2014)^14^
			groES_R	AACCACAAACAGGTGAAGTTATCG		
			groES_HP	[6FAM] TGCCATTTTCAAGCACACGACCTTCA [BHQ1]		
*E. faecium*	*ddl*	U39790,	ddl_F	ACGTAGCATTCTATGATTATGAAGC	124	Naserpour Farivar et al. (2014)^43^
			ddl_R	CATCGTGTAAGCTAACTTCG		
			ddl_P	[6FAM] CAGATTCCAGCCGAAGTGCC [BHQ1]		
*H. influenzae*	*hin*	P26093	Hin_F	CCGGGTGCGGTAGAATTTAATAA	91	Garcha et al. (2012)^44^
			Hin_R	CTGATTTTTCAGTGCTGTCTTTGC		
			Hin_HP	[6FAM]ACAGCCACAACGGTAAAGTGTTCTACG [BHQ1]		
*K. pneumoniae*	*khe*	AF293352	Khe_F	GATGAAACGACCTGATTGCATTC	77	Hartman et al. (2009)^45^
			Khe_R	CCGGGCTGTCGGGATAAG		
			Khe_HP	[6FAM] CGCGAACTGGAAGGGCCCG [BHQ1]		
*M. catarrhalis*	*mor*	U69982	Mor_F	GTGAGTGCCGCTTTTACAACC	72	Greiner et al. (2003)^46^
			Mor_R	TGTATCGCCTGCCAAGACAA		
			Mor_HP	[6FAM] TGCTTTTGCAGCTGTTAGCCAGCCTAA [BHQ1]		
*M. tuberculosis*	*rpo_B*	AL123456	RPOB_FW1	CAAAACAGCCGCTAGTCCTAGTC	84	Devonshire et al. (2015)^47^
			RPOB_RV1	AAGGAGACCCGGTTTGGC		
			RPOB_P1	[6FAM]AGTCGCCCGCAAAGTTCCTCGAA[NFQ]		
*N. meningitidis*	*ctrA*	AM4210808	CtrA_F	GCCGTTTGTTGGCGATATTT	150	O’Sullivan et al. (2014)^14^
			CtrA_R	GCACGAATCACCGACACATT		
			CtrA_HP	[6FAM]CGGTGGTCGGTAAAACGCCTGG [BHQ1]		
*P. aeruginosa*	*regA*	EU342000	regA_F	TGCTGGTGGCACAGGACAT	65	Lee et al. (2006)^48^
			regA_R	TTGTTGGTGCAGTTCCTCATTG		
			regA_MGB	[6FAM] CAGATGCTTTGCCTCAA [MGBNFQ]		
*S. agalactiae*	*sip*	HQ878436	sip_F	ATCCTGAGACAACACTGACA	78	O’Sullivan et al. (2014)^14^
			sip-R	TTGCTGGTGTTTCTATTTTCA		
			sip-HP	[6FAM] ATCAGAAGAGTCATACTGCCACTTC [BHQ1]		
*S. aureus*	*coA*	AB436985	coA_F	GTAGATTGGGCAATTACATTTTGGAGG	117	O’Sullivan et al. (2014)^14^
			coA_R	CGCATCTGCTTTGTTATCCCATGTA		
			coA-HP	[6FAM] TAGGCGCATTAGCAGTTGCATC [BHQ1]		
*S. enterica*	*ttr*	AE006468	ttr_F	CGGCGATGCGTATCACTTT	61	This study
			ttr_R	TTGGACACAGTGCGGTATCC		
			ttr_P	[FAM] CATCGGCATTAACCCGGGCG [BHQ1]		
*S. epidermidis*	*femA_SE*	U23713	F femA-SE	TGCCTTTACAGATAGCATGCCA	172	Francois et al. (2003)^49^
			R femA-SE	AGTAAGTAAGCAAGCTGCAATGACC		
			P femA-SE	TCATTTCACGCAAACTGTTGGCCACTATG		
*S. pneumoniae*	*lytA*	HG531769	LytA_F	ACGCAATCTAGCAGATGAAGC	101	Harris et al. (2008)^50^
			LytA_R	TGTTTGGTTGGTTATTCGTGC		
			LytA_HP	[6FAM] TTTGCCGAAAACGCTTGATACAGGG [BHQ1]		
*S. pyogenes*	*csrR*	JX414161	csrR_F	TGGATGTGGTTGCAGGTTTAGAC	79	O’Sullivan et al. (2014)^14^
			csrR_R	CGGGCAAGTAGTTCTTCAATGG		
			csrR_HP	[6FAM] CGGTGCAGACGACTATATTGTTAAACC [BHQ1]		

### Amplicon sequencing

Amplicon sequencing was performed using two different primer sets; set one targeting variable regions 1 and 2 (V1–2) and set two targeting variable regions 4, 5 and 6 (V4–6) (Table 3). These priming strategies had been previously evaluated as strategy β, employing a combination of forward primers and one single primer in order to increase the specificity of the primer set for V1–2, and strategy γ which used degenerate bases to amplify the V4–6 regions^11^.

**Table 3:** Primers used for this study.

Strategy	Variable Regions	Primer	Sequence (5′–3′)	Position^a^
β	1–2	Forward	GCTCAGATTGAACGCTGGCGG	22–358
			GTTCAGATTGAACGCTGGCGG	
			GCTCAGGACGAACGCTGGCGG	
			GCTCAGGATTAACGCTGGCGG	
			GCTCAGGATGAACGCTGGCGG	
			GCTCAGAATGAACGCTGGCGG	
			GCTCAGGGTGAACGCTGGCGG	
			GCTCAGAGTGAACGCTGGCGG	
		Reverse	ACTGCTGCCTCCCGTAGGAGT	
γ	4–6	Forward	GTGCCAGCAGYYGCGGTAATAC	518–1079
		Reverse	CACRACACGAGCTGACGACA	

^a^Based on numbering from gene *rrsH* accession number AE014075 from *E. coli* CFT073 complete genome NCBI reference sequence: NC_004431.1

Amplicons were prepared of both materials, MCM2α and MCM2β, in triplicate using KAPA HiFi mastermix (Kapa Biosystems) to generate twelve samples for sequencing. Each reaction consisted of 1 × KAPA HiFi Hotstart ReadyMix, 0.3 µM of each primer and each template in a background of 50 ng human gDNA (Promega) in a final volume of 25 µL Nuclease Free Water (Ambion). The reactions were performed on a DNA Engine Tetrad 2 with the following cycling conditions: enzyme activation at 95 °C for 3 min, 30 cycles of denaturation at 98 °C for 15 s, annealing at 72 °C for 15 s and extension at 72 °C for 15 s, a final extension at 72 °C for 5 min and hold at 4 °C. Amplicons were visualised to determine product sizing using the Agilent DNA 1000 kit (Agilent Technologies) version 2.3 on the Agilent Bioanalyzer 2100 Instrument (Agilent Technologies).

Sequencing of the amplicons was performed. In total 12 libraries were prepared using Illumina TruSeq DNA PCR-Free Library Preparation kit processing according to the protocol for 350 bp input size for V1–2 and separately processing the V4–6 amplicons according to the 550 bp insert size (Revision D June 2015). Libraries were indexed using the Nextera indices (Illumina) and were pooled and quantified using KAPA SYBR FAST qPCR Master Mix (2X) Kit (Kapa Biosystems) according to manufacturer’s instructions. Libraries were visualised using a High Sensitivity DNA kit (Agilent Technologies) version 1.03 on the Bioanalyzer 2100. The libraries were diluted to 2 nM and after denaturing diluted to 10 pM. PhiX was spiked in at 5% using 20 pM library which should equate to around 7–10% of total cluster density. DNA sequencing was performed in a single run with an Illumina MiSeq platform using MiSeq V3 reagents (600 cycle chemistry), employing paired-end 300 base reads.

### Shotgun metagenomic sequencing

Shotgun metagenomic sequencing was performed by LGC Genomics GmbH (Berlin, Germany). Libraries were prepared using 25 ng of DNA from three aliquots of MCM2α and MCM2β pools employing an Ovation Rapid DNA Library Preparation Kit (NuGEN). Genomic DNA was sheared to an average size of 400 bp using ultrasonication (Covaris S2 model). Libraries were sequenced on a NextSeq 500 sequencer (Illumina, San Diego, CA, USA) employing paired end 2 × 150 base sequencing. In addition to sequencing of the mixed samples, gDNA from each organism was sequenced as well using the same library preparation and sequencing protocols.

### Data analysis

Paired end sequence reads generated from the shotgun metagenomic DNA libraries were trimmed to remove adapter sequences and bases with quality lower than Phred score 20. Sequences were then assembled using MEGAHIT^37^. Paired end reads were mapped back to the metagenomics assemblies and sequence bins were generated for each sample using metaBAT2^38^. Taxonomy was assigned to individual sequence bins using kraken^39^ for each sample. The relative abundance of each organism was then calculated as the total metagenome length of each unique taxon in base pairs as a percentage of the total number of base pairs sequenced per sample.

### Inter-laboratory study

In total 12 FASTQ data sets were generated from amplicon sequencing consisting of triplicate analysis of each material from sample to sequencing result. Laboratories were invited (Additional File 2) to participate in the analysis of these files using their standard bioinformatics pipelines for 16S rRNA gene amplicon sequence data. If they were in agreement, a link to the data and full description of the study hosted on the following URL: http://pathogenseq.lshtm.ac.uk/mcm.html was provided. They were asked to complete a submission form which collected pertinent information, including: (1) who processed the data, (2) the name of the laboratory, (3) results as biological observation matrices (BIOMs) based on annotation of OTU97 clusters, (4) a description of the analysis to include the command list for the whole bioinformatic process used to produce the results (Additional File 3).

Thirteen participating laboratories returned results. The results were collated in MS Excel 2010, and further analysis carried out using GraphPad Prism 6. The results from the 16S rRNA gene amplicon sequencing from each laboratory had to be normalised to be compared to the dPCR data to take into account that this operon can have different copy numbers depending on the genome. Data from each laboratory was compared to the dPCR analysis of the materials by calculating fold change. To assess agreement between different analytical methods, a cut-off of three-fold difference in relative abundance was applied, as described previously^9^.

The FASTQ files have been deposited in the European Nucleotide Archive (PRJEB34919).

## Results

### Amplicon sequencing

Mock community gDNA from two samples (MCM 2α and β) was PCR-amplified with 16S rRNA gene primers and sequenced using two different strategies. The mean number of reads per sample after sequencing were 845,651 (s.d. 246,478). The performance of these approaches have been previously determined^11^ where they were referred to as strategy β which used multiple forward primers to the same priming site amplifying a region spanning variable regions 1 and 2 and strategy γ which used degenerate bases in the primers to amplify a region spanning variable regions 4, 5 and 6.

### The inter-laboratory study

Thirteen laboratories participated and submitted their results. The analysis steps applied are summarised in Table 4. The full commands used to run pipelines are available in Additional File 4. Most laboratories followed similar approaches except for one laboratory which used the online pipeline BIOiPLUG which implements the EzBioCloud database^40^. It is closed source and so could not be compared to the other pipelines in the same detail. Of the remaining twelve laboratories, eleven performed some form of filtering on the read data before clustering into OTUs and all laboratories merged overlaps between the read pairs. Sequence clustering, OTU assignment and taxonomic assignment differed between laboratories generally by those that used QIIME and mothur. Notable exceptions were the laboratories that used BLAST and USEARCH. There was an almost even split by those that assigned OTUs de novo and those using a closed reference method. Half of the participating laboratories applied further filtering steps to their sequence reads after OTU assignment. The different filtering steps are outlined in Table 4.

**Table 4:** Summary of the methods used by the different laboratories.

Methods	Lab												
	1	2	3	4	5	6	7	8	9	10	11	12	13
Processing of raw reads	Cutadapt—trimmed primers	sickle—qual trim Q35	usearch fastx_truncate—trim primers	None	None	None	None	Trimmomatic—qual trim < 20 Phred score	Trimmomatic—qual trim < 3 Phred score	BIOiPLUG MTB (entirely black box)	None	Primers trimmed. Reads trimmed at Phred quality score < 3. Removed if length < 30 bp	None
Paired end read join	Mothur make.contigs	QIIME1 fastq-join	QIIME1 fastq-join	Mothur make.contigs	Mothur make.contigs	Mothur make.contigs	FLASH	QIIME1 fastq-join	Seqprep	BIOiPLUG MTB	USEARCH fastq_mergepairs	QIIME1 fastq-join	Mothur make.contigs
Removed reads with "N"	Yes	Yes	Yes	Yes	Yes (> 4)	Yes	No	Yes	Yes (> 10%)	BIOiPLUG MTB	No	Yes	Yes
Removed reads by length	Yes (removed outlier small and long reads)	No	No	Yes (removed outlier small and long reads)	Yes (removed outlier long reads)	No	No	No	Removed reads < 100 bp	BIOiPLUG MTB	Removed reads < 250 bp	No	Yes, removed outlier long and short reads
Other pre cluster processing	Removed reads with a homopolymer run longer than 8	Removed sequences that didn't have primer sequence present, truncated reads at Q score < 19	Truncated reads at Q score < 19	Removed reads with a homopolymer run longer than 6, removed sequences that didn't have primer sequence present	Removed reads with a homopolymer run longer than 9	Removed reads with a homopolymer run longer than 8	None	None	None	BIOiPLUG MTB	Removed sequences with greater than 0.5 expected errors, truncated reads at Q15	None	Removed reads with a homopolymer run longer than 6
pre OTU read alignment	Mothur against SILVA database	None	None	None	Mothur against SILVA database	Mothur against SILVA database	None	None	Infernal against RFAM, followed by MAPseq against SILVA	BIOiPLUG MTB	None	None	Mothur against SILVA database
Pre cluster	Mothur ~ 99% similarity	None	None	None	Mothur ~ 99% similarity	Mothur ~ 99% similarity	None	None	None	BIOiPLUG MTB	None	None	Mothur ~ 99% similarity
Chimera detection	VSEARCH	VSEARCH	USEARCH61	USEARCH61	UCHIME	UCHIME	None	USEARCH61	None	BIOiPLUG MTB	None	None	UCHIME
OTU assignment and database	Mothur de novo	QIIME1 UCLUST against SILVA	QIIME1 USEARCH de novo	QIIME1 UCLUST against SILVA	Mothur de novo	Mothur de novo	BLASTN against RefSeq RNA database	QIIME1 USEARCH de novo	MAPseq against SILVA	BIOiPLUG MTB	USEARCH de novo OTU clustering	UCLUST OTU clustering against Greengenes. USEARCH de novo clustering against failures	Mothur de novo
Taxonomic classifcation and database	Mothur against RDP database	QIIME1 UCLUST against SILVA	QIIME1 RDP against SILVA	QIIME1 PYNAST aginst SILVA	Mothur against custom database	Mothur against SILVA database	BLASTN against RefSeq RNA database	QIIME1 UCLUST against SILVA	MAPseq against SILVA	BIOiPLUG MTB	QIIME1 UCLUST against Greengenes	QIIME1 UCLUST against Greengenes	Mothur against SILVA database
Post-processing	Sequences removed if less than 90 reads in 50% of samples	OTUs not represented in all 3 repeats were discarded	QIIME1 removed sequences in table present at fraction 0.005 of total number of reads	QIIME1 removed sequences in table present at fraction 0.001 of total number of reads	None	Oligotyping and ARB	None	QIIME1 removed sequences in table present at fraction 0.00005 of total number of reads	None	BIOiPLUG MTB	Took forward only OTUs with species assignment	None	OTUs kept where mean relative abundance within replicates > 0.0001
Normalisation of reads	Percentage abundance per sample	Percentage abundance per sample	Percentage abundance per sample	Percentage abundance per sample	Percentage abundance per sample	Percentage abundance per sample	Percentage abundance per sample	CSS	Percentage abundance per sample	BIOiPLUG MTB	Percentage abundance per sample	Percentage abundance per sample	Percentage abundance per sample

**Figure 1:**
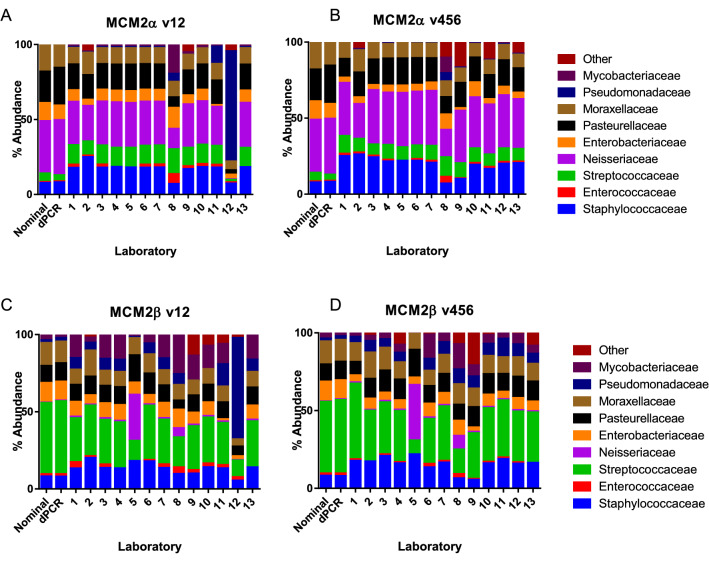
(**A**–**D**) The % family abundance reported by each laboratory including the nominal and dPCR reported composition for the two materials; MCM2α, variable regions V1–2 (**A**) and V4–6 (**B**) and MCM2β, variable regions, V1–2 (**C**) and V4–6 (**D**).

Results were reported as relative abundance with 97% OTU identity by the participating laboratories. As the taxonomic depth reported by the laboratories varied, it was decided that in order to compare the data the approach which would use the largest proportion of the data for cross-comparison was chosen; comparing the results using family level was therefore the optimal approach (Fig. 1A–D).

### 16S rRNA gene amplicon sequencing compared to dPCR results

The relative abundance of each taxonomic group was determined using 16S rRNA gene amplicon sequence data, and these relative abundance (RA) values were compared to the ‘true’ relative abundance, as determined through dPCR analysis of each taxon independently.

The dPCR approach in this study characterised the material according to single copy species specific genes whereas the 16S rRNA gene amplicon sequencing results have to be normalised based on the number of copies of this operon per genome. Differences are reported as fold-differences in RA between 16S amplicon results and dPCR results. In general, the results of the 16S rRNA amplicon sequencing approach differed by less than three-fold when compared to the dPCR value (Additional File 5). Differences greater than three-fold were described by 11/13 laboratories when reporting the abundance of Mycobacteriaceae (range 5.70–94.72 fold) and 10/13 laboratories when reporting the abundance of Pseudomonadaceae (range 3.26–466.34) in MCM2α v12. These families of organisms were the least abundant organisms in the sample; Pseudomonadaceae at 0.0015% and Mycobacteriaceae at 0.002% according to dPCR (Fig. 1). To determine if this observation was as a result of these families being the least abundant in the material, and therefore might represent a threshold which could be applied when analysing data for composition, we compared the results observed for the same variable regions but with the second mock DNA sample (MCM2β). This time, a different pattern was observed. The reported abundance of Neisseriaceae (the least abundant family of organisms, present at 0.005%) was very similar to the dPCR result, differing only by onefold on average except for laboratories 5 and 8 which differed by 61- and 11-fold respectively. Laboratory 5 used a custom database for taxonomic assignment, whereas the other laboratories used large public databases (Table 4). Laboratory 8 was the only participant to normalise their data using CSS, rather than taking a fraction from the total number of sequences within a sample. For most other laboratories, differences were less than three-fold. The differences comparing V1–2 amplicon sequencing to dPCR of MCM2β were greatest for the Mycobacteriaceae with 9/13 laboratories reporting fold differences greater than three.

When analysing the sequence data from V4–6 of MCM2α, all differences were less than three-fold compared to dPCR. For MCM2β differences in general were also less than three-fold except for large differences observed by laboratory 8 in terms of abundance of Mycobacteriaceae and Neisseriaceae (6.41 and 17.43 fold), Mycobacteriaceae reported by laboratory 6 (6.14 fold) in MCM2α (5, 33 and 49 fold respectively) and Neisseriaceae reported by laboratory 5 (73.29 fold).

In general, it was observed that the V4–6 region gave the most similar relative abundance compared to the dPCR results. It was observed for this primer set that many more OTUs were generated (as an example laboratory 13 reported an average 34 OTUs for the V1–2 region compared to 3240 OTUs for the V4–6 region). This could be due to the fact the V4–6 generates a much larger amplicon (~ 564 bp amplicon size for V4–6 v ~ 337 bp amplicon size for V1–2) and because of this could have a higher expected high error rate within those sequences which may lead to an over estimation of OTU richness.

**Figure 2:**
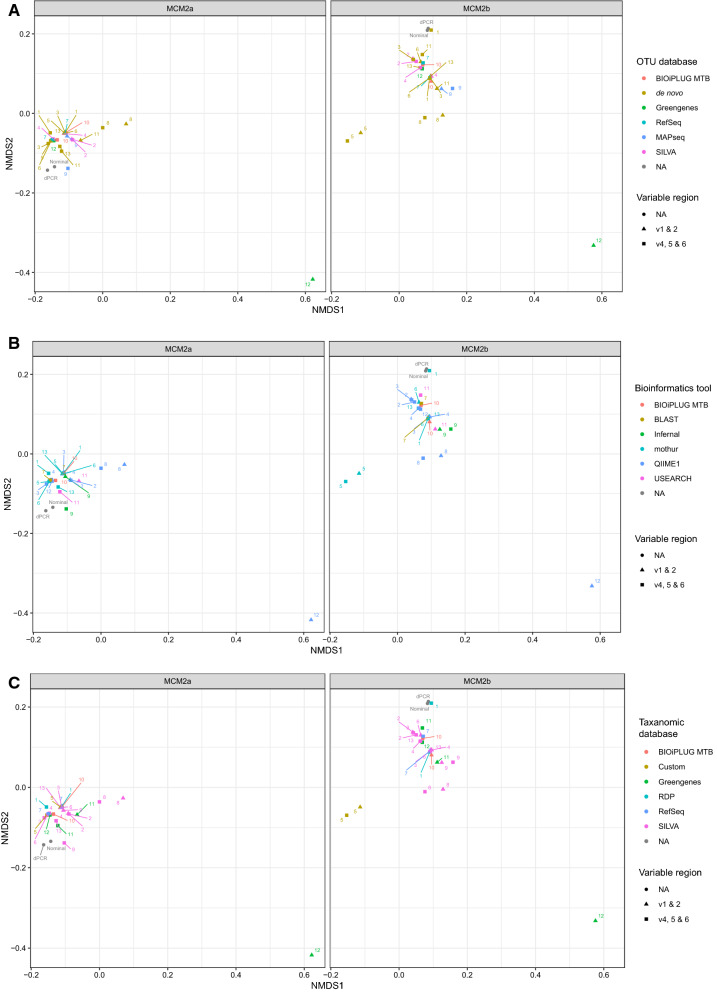
(**A**–**C**) NDMS (non-metric multidimensional scaling) plots to see if results from the 13 laboratories cluster according to OTU assignment tool (**A**), OTU assignment database (**B**) and taxa assignment database (**C**). OTU assignment database was included for laboratories that used closed reference OTU picking. They generally compared their sequences against a reference database of sequences that clustered the reads into OTUs based on sequence similarity. Later, many laboratories assigned taxonomic identifiers to each of these OTUs using a separate database which had sequence data (sometimes not clustered into OTUs) and which taxa that sequence originated from.

To determine the influence that the different steps in the bioinformatic pipeline had on the results, results were clustered using NDMS (non-metric multidimensional scaling) plots to see if results group according to OTU assignment tool, OTU assignment database and taxa assignment database (Fig. 2A–C). Using ANOSIM we found statistically significant clustering of results due to the variable region amplified (*p* = 0.027) but not by laboratory, OTU assignment tool and taxa assignment database. The choice of OTU database or de novo methods also showed statistically significant clustering (*p* = 0.021). The choice of the Greengenes database for this step had a significant influence on the results, as observed by the data reported from laboratory 12 (Fig. 2A). This database choice however did not influence the results to this degree when laboratory 12 analysed V4–6 of both materials. In addition, results from laboratory 8 were more different compared to the other laboratories in the results reported for MCM2α and MCM2β perhaps due to normalisation strategy. It was observed that the results reported by laboratory 5 for MCM2β for V1–2 and V4–6 differed compared to the other laboratories due to using a custom database for taxa assignment rather than the choice of OTU database or assignment tool (Fig. 2C).

Some of the laboratories did not report the presence of some of families of organisms (Additional File 5). Enterococcaceae appeared to be the family most commonly unreported; by laboratory 4 in V1–2 of both materials, laboratory 5 in V4–6 of both materials, laboratory 13 in MCM2α V1–2 and MCM2β V4–6 and laboratory 2 in MCM2β V4–6. In addition, Mycobacteriaceae were not reported by laboratory 12 in V1–2 of both materials and Psuedomonadaceae were not reported by laboratory 5 in V4–6 of both materials.

### Shotgun metagenomic sequencing compared to dPCR results

Shotgun metagenomic sequencing was performed on each of the gDNAs in addition to triplicate sequencing runs on the MCM2α and MCM2β (Additional File 6). The mean number of reads per sample after sequencing were 27,678,330 (s.d. 5,540,768). After metagenomic assembly of three MCM2α and three MCM2β replicates the average percentage of reads that mapped to the assembled contigs were 87%, 88% and 86% for MCM2α and 72%, 89% and 90% for MCM2β. The depth of sequencing of the assembled organisms in each sample differed with their relative abundance and ranged from a mean of 16 reads in low abundance organisms up to a mean of 900 reads in the high abundance samples. When compared to the dPCR data it was observed that the results were less than 1.8-fold different in terms of abundance of each organism between the two methods except for *P. aeruginosa* which was over-represented in both materials according to shotgun metagenomics sequencing (7.34 higher in MCM2α and 5.59 in MCM2β) (Additional File 7). Previous work analysing a different material had the same observation, but the underlying reason is not fully understood^11^. The source of *P. aeruginosa* was the same between the two studies but different batches were used. The shotgun sequencing method did not identify organisms present at the lowest abundance (*S. pneumoniae* and *A. baumannii* in MCM2α and *S. enterica*, *A. baumannii* and *N. meningitidis* in MCM2β). These organisms were of low abundance in the materials (≤ 0.003%) and so this could be explained by the sequencing coverage not being adequate to identify organisms at this level of abundance.

Additional taxa reported in the shotgun metagenomics data included Bradyrhizobiaceae and *Bradyrhizobium*, each present at 0.08%. These taxa were reported in all three units analysed. When each individual genomic DNA preparation was analysed by shotgun sequencing, this organism was present in the genomic preparation of hCMV (14% of total dataset), *K. pneumoniae* (12%) and *S. aureus* (0.03%). The Bradyrhizobiaceae family was also observed in the 16S rRNA analysis of V4–6 of MCM2α and MCM2β by laboratory 5 at 0.01% and 0.001% respectively. Laboratories 7, 9, 10 and 12 also reported this family but at a much lower abundance (< 0.00001%) and this was observed from both V4–6 and V1–2. For laboratories reporting Bradyrhizobiaceae presence it was not observed in all sequencing replicates unlike what was reported for the shotgun sequencing data. This family of bacteria has been observed as a contaminant in previous next generation sequencing studies^41,42^.

## Discussion

The 16S rRNA gene amplicon sequencing procedure requires a multi-step process in order to determine the microbial composition of a sample. Bioinformatic analysis is crucial and involves multiple tools and steps as well as varying parameters. The bioinformatic tools used in this study represent relatively straightforward and simple to integrate for 16S rRNA gene amplicon analysis and are relatively less computationally intensive than when studying entire metagenomes. With the increasing diversity of bioinformatics tools and of the parameters which determine how each of these tools are used, it can be difficult to determine which tool will give the most accurate representation of the sample composition. In the face of this growing challenge, the use of control materials aids researchers in pipeline validation and choice of pipeline. These materials can also be used to evaluate current and future software versions. However, the choice of the most appropriate control material is itself an unresolved challenge and the ‘true’ composition of the reference community can be difficult to verify. Here we applied dPCR to determine the absolute and relative abundance of each organism in our control materials using species-specific assays that allowed for an accurate measurement of the composition of each of the materials and provides an absolute method for determining the composition of microbial standards.

In this study it was observed that in general there was good agreement when comparing the material composition according to the different 16S rRNA gene amplicon sequencing data results from the different laboratories to dPCR, with differences of less than three fold. Most large discrepancies were encountered with organisms present at low abundance. However, this result varied by analysis of variable regions under investigation. For example, analysis of 16S rRNA gene amplicon sequence data generated with the V4–6 primer set was more concordant with the dPCR analysis of the composition of the source materials than were amplicons covering the V1–2 region. However the method using this primer set as was observed for laboratory 5 was not concordant with the dPCR result. This laboratory used a custom database for taxonomic assignment, whereas the other laboratories used large public databases. It could be that the custom database was missing some key sequences that would lead to under-representation and over-representation of certain taxa.

Some of the laboratories did not identify families of organisms known to be present in the materials. This must have been because of the different methods applied. Laboratory 4, for example, applied a very stringent 0.1% relative abundance OTU cut-off which would remove many of the OTUs present, including the Enterococcaceae. It was observed that it was always the lowest abundance taxa that were found to be missing from the results when stringent filtering of the data was applied. The shotgun metagenomic sequencing approach also under-estimated the presence of this family. This could be due to the fact that this family is present at 0.6% in the MCM2α. Both 16S rRNA gene amplicon and metagenomic sequencing find it hard to differentiate between low abundance organisms and low-level contamination of bacterial DNA in each sample.

The inclusion of technical replicates allowed the reproducibility of the individual laboratory methods to be investigated. Overall they demonstrated good precision with coefficient of variation (CV) of < 10% in general (Table 5), apart from the results from laboratory 12 which, although used QIIME 1 like many others, performed no filtering of the OTU assigned reads which have previously been shown to be of poorer quality.

**Table 5:** The precision of MCM2α reported as percentage coefficient of variation (%CV) from triplicate technical repeats of each of the family of organisms reported by the 13 different laboratories for the 16S rRNA sequencing approach of MCM2α V12 (A), MCM2α V4–6 (B), MCM2β V1–2 (C), MCM2β V4–6 (D), and of the shotgun metagenomic sequencing approach for MCM2α (E) and MCM2β (F).

Family	Laboratory number (% CV)											
	1	3	4	5	6	7	8	9	10	11	12	13
**(A)**												
Neisseriaceae	1.2	1.0	1.1	1.1	1.1	1.1	0.4	1.1	0.9	0.9	104.0	1.1
Pasteurellaceae	1.9	2.0	2.2	2.1	2.1	2.0	0.6	2.1	2.8	1.9	13.9	2.0
Moraxellaceae	3.5	3.1	2.6	3.5	2.9	2.9	0.9	2.8	3.6	3.0	17.4	2.8
Enterobacteriaceae	1.3	1.8	1.7	1.3	1.8	1.8	0.9	2.1	0.4	2.3	31.4	1.8
Staphylococcaceae	2.4	2.4	2.2	2.7	2.4	2.3	0.6	2.3	2.4	2.3	6.8	2.4
Streptococcaceae	3.7	3.6	3.7	3.6	3.7	3.7	0.9	3.8	3.5	3.6	38.5	3.8
Enterococcaceae	2.5	2.4	0.0	0.1	2.4	2.4	1.3	2.6	3.0	2.1	50.9	0.0
Mycobacteriaceae	7.5	8.5	17.0	9.4	8.4	8.4	1.4	7.8	6.5	9.4	0.0	8.5
Pseudomonadaceae	2.0	1.9	0.3	2.1	1.3	1.7	0.5	7.0	71.3	1.8	4.1	1.5
**(B)**												
Neisseriaceae	6.24	2.59	2.82	5.65	2.91	0.94	0.66	2.22	3.37	3.30	2.39	2.75
Pasteurellaceae	10.58	3.58	1.82	4.45	1.85	2.41	0.77	0.97	1.11	3.02	1.69	1.99
Moraxellaceae	8.03	3.96	6.18	9.76	6.00	3.38	1.41	5.69	5.01	5.50	5.29	6.33
Enterobacteriaceae	5.23	2.22	1.63	4.83	1.97	0.57	0.96	2.09	8.13	2.02	1.37	2.24
Staphylococcaceae	1.57	1.23	1.27	3.22	1.23	0.54	0.91	1.79	1.34	0.36	1.31	1.31
Streptococcaceae	7.04	4.24	4.60	9.43	4.60	3.34	0.66	4.96	3.57	3.88	4.08	4.51
Enterococcaceae	28.15	9.26	11.20	0.00	12.91	9.85	2.22	14.90	12.16	10.85	12.14	13.09
Mycobacteriaceae	11.39	7.95	0.23	29.59	16.88	10.96	1.81	83.48	21.69	11.64	12.72	13.35
Pseudomonadaceae	8.73	12.55	0.23	0.00	11.27	8.32	4.10	9.61	11.80	6.06	8.61	10.99
**(C)**												
Streptococcaceae	0.61	1.32	1.28	3.57	3.87	1.06	0.59	0.99	0.58	1.02	24.52	1.17
Moraxellaceae	4.60	4.65	4.54	3.54	6.71	4.88	1.09	5.06	4.53	4.63	18.70	4.79
Enterobacteriaceae	1.39	3.96	0.92	1.25	3.82	1.48	0.60	1.60	3.00	3.55	8.39	1.45
Pasteurellaceae	2.68	1.83	1.95	2.07	7.14	1.76	0.68	1.86	1.38	1.85	21.20	1.84
Staphylococcaceae	3.77	4.28	4.03	2.69	4.42	3.99	0.65	39.95	3.72	4.05	17.94	4.07
Pseudomonadaceae	5.36	4.60	4.35	2.11	2.84	4.82	1.14	5.63	4.43	3.79	5.96	4.64
Enterococcaceae	6.59	5.63	0.00	0.06	4.92	5.45	1.72	5.46	5.17	5.01	20.47	4.30
Mycobacteriaceae	1.72	1.36	1.41	9.39	5.15	1.46	0.65	1.25	1.64	1.24	0.00	1.26
Neisseriaceae	3.30	1.80	0.29	1.11	4.02	1.50	2.97	1.60	3.55	2.30	126.69	1.50
**(D)**												
Streptococcaceae	5.46	4.27	4.13	9.26	2.81	3.18	0.66	4.90	3.90	4.16	4.25	4.57
Moraxellaceae	11.77	6.49	5.52	10.10	3.57	6.00	0.20	5.84	6.27	5.63	6.18	5.93
Enterobacteriaceae	7.04	3.96	4.19	5.08	1.64	3.88	0.96	2.58	4.79	3.24	3.80	3.85
Pasteurellaceae	18.45	5.55	6.73	4.76	3.59	5.29	0.93	6.53	6.37	5.61	6.22	8.84
Staphylococcaceae	8.52	3.74	3.83	3.43	5.42	3.24	0.60	85.54	4.46	3.05	3.95	5.00
Pseudomonadaceae	7.21	1.58	3.47	0.00	3.69	1.99	1.27	2.18	2.36	3.45	2.59	2.36
Enterococcaceae	9.53	3.07	5.54	0.00	7.06	2.56	1.70	3.71	5.51	1.18	3.15	0.00
Mycobacteriaceae	1.25	2.67	4.81	29.76	1.02	3.20	0.86	4.57	2.73	5.83	4.09	4.51
Neisseriaceae	6.64	1.35	0.74	5.32	2.97	3.26	3.38	3.02	1.45	4.53	3.98	3.48

Organism	% CV											
(**E**)												
*N. meningitidis*	1.59											
*K. pneumoniae*	1.72											
*H. influenzae*	3.72											
*M. catarrhalis*	4.44											
*S. aureus*	8.92											
*S. pyogenes*	8.15											
*E. coli*	12.62											
*S. agalactiae*	10.96											
*P. aeruginosa*	21.80											
*E. faecalis*	12.97											
*M. tuberculosis*	42.92											
*S. enterica*	*											
*S. pneumoniae*	NR											
*A. baumanii*	NR											
**(F)**												
*S. pneumoniae*	12.39											
*P. aeruginosa*	16.96											
*M. catarrhalis*	5.63											
*M. tuberculosis*	13.69											
*H. influenzae*	11.98											
*S. agalactiae*	52.67											
*S. aureus*	8.28											
*K. pneumoniae*	58.80											
*E. coli*	2.73											
*E. faecium*	8.56											
*S. pyogenes*	3.19											
*S. epidermidis*	31.43											
*S. enteritidis*	NR											
*A. baumanii*	NR											
*N. meningitidis*	NR											

The data is ordered by the most abundant organisms. Data from Laboratory 2 was not included as individual data was not reported, instead mean % abundance was reported.

*NR* not reported.

*Reported in one repeat.

In this setting it was observed that the bioinformatic analysis of the V4–6 of 16S rRNA gene more closely resembled the determined composition using dPCR with V4–6 mean Bray–Curtis similarity to dPCR of 0.62 (95% CI 0.56–0.69) compared to 0.58 (95% CI 0.51–0.65) for V1–2. After omission of the results from laboratory 8 for MCM2α, all of the other pipelines were on average 0.84 fold different compared to dPCR (range 0.02—2.4). This is a very impressive result in terms of performance of the various 16S rRNA pipelines, all of which are composed of multiple steps, in analysing these materials.

Shotgun metagenomic sequencing results compared to dPCR results demonstrated for the most part good agreement, except for reporting of the abundance of *P. aeruginosa* where there were the largest differences, and also in the reporting of the lower abundance organisms in the materials. The precision of this approach was also determined and was demonstrated to be very good except for the lower abundance organisms (present at ≤ 0.01% abundance). It should be noted here that the comparison to the shotgun metagenomic sequencing approach was not from an inter-laboratory study, as was the case for the 16S rRNA data, but was from a single analysis workflow. So a further study is warranted to compare analysis tools for shotgun metagenomic data.

## Conclusions

Determining the microbial composition of a sample can be undertaken by various high-throughput sequencing. Frequently this involves sequencing of variable regions of the 16S rRNA genes. We evaluated the multi-step process in assigning OTUs to a complex sample in terms of repeatability and reproducibility using control materials containing complex communities of microbes. The methods in general demonstrated high precision; however caution needs to be applied when drawing conclusions for microbiome data as variation between methods could significantly alter results.

In this study the reproducibility of the bioinformatics component was optimal when analysing the V4–6 regions which gave the most concordance with the dPCR analysis and the sequencing approach. While there was good agreement in general when comparing the different bioinformatics approaches, caution is required when using custom databases and applying high-stringency cut-offs that could misrepresent the relative abundance of organisms present. These findings are independent of software versions used and should be considered for current and future formats. This study provides compelling evidence of the importance of interrogating methods through the use of carefully designed control materials which could underpin future selection of the most appropriate methods to be applied to samples of interest.

## Supplementary Information

Supplementary Information 1.

Supplementary Information 2.

Supplementary Information 3.

Supplementary Information 4.

Supplementary Information 5.

Supplementary Information 6.

Supplementary Information 7.

## Data Availability

The datasets generated and/or analysed during the current study are available in the European Nucleotide Archive repository, https://www.ebi.ac.uk/ena/browser/view/PRJEB34919.

## Abbreviations

OTU: Operational taxonomic unit
dPCR: Digital PCR
QIIME: Quantitative insights into microbial ecology
MG-RAST: Metagenomics rapid annotations using subsystems technology
MIxS: Minimum information about any (X) sequence
WGS: Whole genome sequencing
GMI: Global microbial identifier
EMQN: European molecular genetics quality network
EQA: External quality assessment
MCM: Metagenomic control material
gDNA: Genomic DNA
NDMS: Non-metric multidimensional scaling
