# Supplementary material for: An inter-laboratory study to investigate the impact of the bioinformatics component on microbiome analysis using mock communities
Source: Sci Rep. 2021 May 19;11:10590. doi: 10.1038/s41598-021-89881-2 (PMC8134577; doi:10.1038/s41598-021-89881-2)
Supplement: Supplementary file 1 — Supplementary Information 1. [file 41598_2021_89881_MOESM1_ESM.docx]

An inter-laboratory study to investigate the impact of the bioinformatics component on microbiome analysis using mock communities

Denise M. O’Sullivan, Ronan M. Doyle, Sasithon Temisak, Nicholas Redshaw, Alexandra S. Whale, Grace Logan, Jiabin Huang, Nicole Fischer, Gregory C. A. Amos, Mark D. Preston, Julian R. Marchesi, Josef Wagner, Julian Parkhill, Yair Motro, Hubert Denise, Robert D. Finn, Kathryn A. Harris, Gemma L. Kay, Justin O’Grady, Emma Ransom-Jones, Huihai Wu, Emma Laing, David J. Studholme, Ernest Diez Benavente, Jody Phelan, Taane G. Clark^,^, Jacob Moran-Gilad, Jim F. Huggett

**Additional File 1: Digital MIQE checklist for authors, reviewers and editors**

| ITEM TO CHECK | IMPORTANCE | COMMENTS |
| --- | --- | --- |
| EXPERIMENTAL DESIGN |  |  |
| Definition of experimental and control groups | E | Methods |
| Number within each group | E | Methods |
| Assay carried out by core lab or investigator's lab? | D | At core laboratory |
| Power analysis | D | Not included |
| SAMPLE |  |  |
| Description | E | Methods |
| Volume or mass of sample processed | E | N/A |
| Microdissection or macrodissection | E | N/A |
| Processing procedure | E | N/A |
| If frozen - how and how quickly? | E | N/A |
| If fixed - with what, how quickly? | E | N/A |
| Sample storage conditions and duration (especially for FFPE samples) | E | N/A |
| NUCLEIC ACID EXTRACTION |  |  |
| Quantification - instrument/method | E | Methods |
| Storage conditions: temperature, concentration, duration, buffer | E | Methods |
| DNA or RNA quantification | E | Methods |
| Quality/integrity-instrument/method; e.g. RIN/RQI and trace or 3’:5’ | E | N/A |
| Template structural information | E | N/A |
| Template modification (digestion, sonication, pre-amplification etc.) | E | N/A |
| Template treatment (initial heating or chemical denaturation) | E | N/A |
| Inhibition dilution or spike; | E | N/A |
| DNA contamination assessment of RNA sample | E | N/A |
| Details of DNase treatment where performed | E | N/A |
| Manufacturer of reagents used and catalogue number | D | Methods |
| Storage of nucleic acid: temperature, concentration, duration, buffer | E | Methods |
| REVERSE TRANSCRIPTION (If necessary) |  |  |
| cDNA priming method + concentration | E | N/A |
| One or two step protocol | E | N/A |
| Amount of RNA used per reaction | E | N/A |
| Detailed reaction components and conditions | E | N/A |
| RT efficiency | D | N/A |
| Estimated copies measured with and without addition of RT* | D | N/A |
| Manufacturer of reagents used and catalogue number | D | N/A |
| Reaction volume (for two step reverse transcription reaction) | D | N/A |
| Storage of cDNA: temperature, concentration, duration, buffer | D | N/A |
| dPCR TARGET INFORMATION |  |  |
| Sequence accession number | E | Table 2 |
| Location of amplicon | D | N/A |
| Amplicon length | E | Table 2 |
| In silico specificity screen (BLAST, etc) | E | Upon request |
| Pseudogenes, retropseudogenes or other homologs? | D | N/A |
| Sequence alignment | D | Not included |
| Secondary structure analysis of amplicon and GC content | D | Not included |
| Location of each primer by exon or intron (if applicable) | E | N/A |
| Where appropriate, which splice variants are targeted? | E | N/A |
| dPCR OLIGONUCLEOTIDES |  |  |
| Primer sequences and/or amplicon context sequence** | E | Table 2 |
| RTPrimerDB Identification Number | D | N/A |
| Probe sequences** | D | Table 2 |
| Location and identity of any modifications | E | Table 2 |
| Manufacturer of oligonucleotides | D | N/A |
| Purification method | D | N/A |
| dPCR PROTOCOL |  |  |
| Complete reaction conditions | E | Previously described in O’Sullivan et al. 2014 |
| Reaction volume and amount of RNA/cDNA/DNA | E | Previously described in O’Sullivan et al. 2014 |
| Primer, (probe), Mg++ and dNTP concentrations | E | Previously described in O’Sullivan et al. 2014 |
| Polymerase identity and concentration | E | Proprietary |
| Buffer/kit Catalogue No and manufacturer | E | Previously described in O’Sullivan et al. 2014 |
| Exact chemical constitution of the buffer | D | Proprietary |
| Additives (SYBR Green I, DMSO, etc.) | E | Proprietary |
| Plates/tubes Catalogue No and manufacturer | D | Not included |
| Complete thermal cycling parameters | E | Previously described in O’Sullivan et al. 2014 |
| Reaction setup | D | Manual |
| Gravimetric or volumetric dilutions (manual/robotic) | D | Manual, volumetric |
| Total PCR reaction volume prepared | D | Previously described in O’Sullivan et al. 2014 |
| Partition number | E | Previously described in O’Sullivan et al. 2014 |
| Individual partition volume | E | Previously described in O’Sullivan et al. 2014 |
| Total volume of the partitions measured (effective reaction size) | E | Previously described in O’Sullivan et al. 2014 |
| Partition volume variance/standard deviation | D | Not determined |
| Comprehensive details and appropriate use of controls | E | Previously described in O’Sullivan et al. 2014 |
| Manufacturer of dPCR instrument | E | Method section and previously described in O’Sullivan et al. 2014 |
| dPCR VALIDATION |  |  |
| Optimisation data for the assay | D | Previously described in O’Sullivan et al. 2014 |
| Specificity (when measuring rare mutations, pathogen sequences etc.) | E | Previously described in O’Sullivan et al. 2014 |
| Limit of detection of calibration control | D | Not provided |
| If multiplexing, comparison with singleplex assays | E | N/A |
| DATA ANALYSIS |  |  |
| Average copies per partition (λ or equivalent ) | E | Previously described in O’Sullivan et al. 2014 |
| dPCR analysis program (source, version) | E | Previously described in O’Sullivan et al. 2014 |
| Outlier identification and disposition | E | Not provided |
| Results of NTCs | E | Previously described in O’Sullivan et al. 2014 |
| Examples of positive(s) and negative experimental results as supplemental data | E | Previously described in O’Sullivan et al. 2014 |
| Where appropriate, justification of number and choice of reference genes | E | Previously described in O’Sullivan et al. 2014 |
| Where appropriate, description of normalisation method | E | Previously described in O’Sullivan et al. 2014 |
| Number and concordance of biological replicates | D | Not provided |
| Number and stage (RT or qPCR) of technical replicates | E | Previously described in O’Sullivan et al. 2014 |
| Repeatability (intra-assay variation) | E | Previously described in O’Sullivan et al. 2014 |
| Reproducibility (inter-assay/user/lab etc. variation ) | D | Upon request |
| Experimental variance or confidence interval*** | E | Previously described in O’Sullivan et al. 2014 |
| Statistical methods used for analysis | E | Previously described in O’Sullivan et al. 2014 |
| Data submission using RDML | D | Not provided |

* Assessing the absence of DNA using a no RT assay (or where RT has been inactivated) is essential when first extracting RNA. Once the sample has been validated as DNA-free, inclusion of a no-RT control is desirable, but no longer essential.

** Disclosure of the primer and probe sequence is highly desirable and strongly encouraged. However, since not all commercial pre-designed assay vendors provide this information when it is not available assay context sequences must be submitted (48)

*** When single dPCR experiments are performed, the variation due to counting error alone should be calculated from the binomial (or suitable equivalent) distribution.
