# Supplementary material for: An inter-laboratory study to investigate the impact of the bioinformatics component on microbiome analysis using mock communities
Source: Sci Rep. 2021 May 19;11:10590. doi: 10.1038/s41598-021-89881-2 (PMC8134577; doi:10.1038/s41598-021-89881-2)
Supplement: Supplementary file 2 — Supplementary Information 2. [file 41598_2021_89881_MOESM2_ESM.docx]

An inter-laboratory study to investigate the impact of the bioinformatics component on microbiome analysis using mock communities

Denise M. O’Sullivan, Ronan M. Doyle, Sasithon Temisak, Nicholas Redshaw, Alexandra S. Whale, Grace Logan, Jiabin Huang, Nicole Fischer, Gregory C. A. Amos, Mark D. Preston, Julian R. Marchesi, Josef Wagner, Julian Parkhill, Yair Motro, Hubert Denise, Robert D. Finn, Kathryn A. Harris, Gemma L. Kay, Justin O’Grady, Emma Ransom-Jones, Huihai Wu, Emma Laing, David J. Studholme, Ernest Diez Benavente, Jody Phelan, Taane G. Clark^,^, Jacob Moran-Gilad, Jim F. Huggett

Additional File 2: Invitation for the inter-laboratory study.

<http://pathogenseq.lshtm.ac.uk/mcm.html>
