# Supplementary material for: An inter-laboratory study to investigate the impact of the bioinformatics component on microbiome analysis using mock communities
Source: Sci Rep. 2021 May 19;11:10590. doi: 10.1038/s41598-021-89881-2 (PMC8134577; doi:10.1038/s41598-021-89881-2)
Supplement: Supplementary file 3 — Supplementary Information 3. [file 41598_2021_89881_MOESM3_ESM.docx]

An inter-laboratory study to investigate the impact of the bioinformatics component on microbiome analysis using mock communities

Denise M. O’Sullivan, Ronan M. Doyle, Sasithon Temisak, Nicholas Redshaw, Alexandra S. Whale, Grace Logan, Jiabin Huang, Nicole Fischer, Gregory C. A. Amos, Mark D. Preston, Julian R. Marchesi, Josef Wagner, Julian Parkhill, Yair Motro, Hubert Denise, Robert D. Finn, Kathryn A. Harris, Gemma L. Kay, Justin O’Grady, Emma Ransom-Jones, Huihai Wu, Emma Laing, David J. Studholme, Ernest Diez Benavente, Jody Phelan, Taane G. Clark^,^, Jacob Moran-Gilad, Jim F. Huggett

Additional File 3: Submission form for participants for the inter-laboratory study.


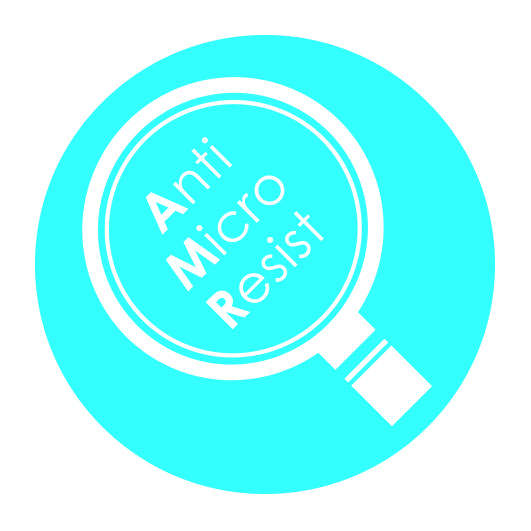


**Comparing 16S rRNA sequence data analytical pipelines for microbiome characterisation: A multi-laboratory study**

**Submission Form**

1. Institute Name
2. Department Name
3. Name of person responsible for analysis
4. Description of bioinformatic process.

Note: Please include a separate file with the command list for the whole bioinformatic process which was used to produce the results

1. Report results as % relative abundance (97 % identity)
